# PHYSIOLOGICAL FRACTURE HEALING IS UNAFFECTED BY NEUTROPHIL-DERIVED IL-6 OR IL-6R SIGNALING IN MICE

**DOI:** 10.1097/SHK.0000000000002615

**Published:** 2025-05-14

**Authors:** Verena Fischer, Oliver Küppers, Lena Steppe, Benjamin Thilo Krüger, Juan Hidalgo, Melanie Haffner-Luntzer, Anita Ignatius

**Affiliations:** 1 Institute of Orthopedic Research and Biomechanics, Ulm University Medical Center, Ulm, Germany; 2 Institute of Neurosciences and Dept. Cellular Biology, Physiology, and Immunology, Universitat Autònoma de Barcelona, Barcelona, Spain

**Keywords:** Neutrophils, interleukin-6, interleukin-6 receptor signaling, bone fracture healing, inflammation

## Abstract

**Background**: Neutrophils are the predominant immune cell type in the early fracture hematoma, playing key roles in orchestrating the immune response and bone repair by clearing pathogens and debris, producing extracellular traps and proteases, and releasing various signaling molecules. However, neutrophil roles in fracture healing remain incompletely understood. They are a key source of interleukin-6 (IL-6) and the soluble IL-6 receptor (sIL-6R), driving both IL-6 classic signaling *via* membrane-bound IL-6R and trans-signaling *via* sIL-6R. Classic signaling drives neutrophil infiltration into the fracture hematoma and is crucial for bone repair, whereas trans-signaling impairs healing after severe trauma. Here, we examined neutrophil-specific IL-6 signaling in fracture healing. **Methods**: We used male mice with neutrophil-specific deletion of *IL-6* or *IL-6R*. Physiological bone phenotype and effects on fracture healing (external fixator stabilized femur osteotomy) were assessed in 12-week-old mice by flow cytometry, cytokine multiplex analysis, biomechanical testing, micro-computed tomography, and histomorphometry. **Results**: While neutrophil-specific deletion of *IL-6* or *IL-6R* did not affect bone under physiological conditions, *IL-6R* deletion led to a reduction in neutrophils and macrophages and an increase in T lymphocytes. The immune response to fracture was unaffected by either deletion, because cytokine levels in the fracture hematoma and serum remained unchanged compared to controls after 6 h. Additionally, the biomechanical properties of fractured femurs together with structural and cellular bone parameters on day 21 did not differ compared to controls. **Conclusions**: Neutrophil-induced IL-6 signaling appears nonessential for physiological bone turnover and fracture healing. Its role in impaired healing under conditions of excessive inflammation remains to be determined.

## INTRODUCTION

Fracture healing is a complex biological process characterized by overlapping phases of inflammation, repair, and remodeling, which involves a finely tuned interplay between various immune cells and cytokines (1). Neutrophils are the predominant cell type in the early fracture hematoma (2–6). Despite their abundance, their role and effector mechanisms in bone repair remain less well defined compared to other immune cells such as macrophages, mast cells and lymphocytes. Neutrophils engulf pathogens, form neutrophil extracellular traps (NETs), and release reactive oxygen species, various proteases (e.g., elastase, myeloperoxidase), and numerous inflammatory mediators (7), all of which play crucial roles in regulating the early inflammatory phase and subsequent bone healing. Previous studies showed that neutrophils contribute to the resolution of inflammation, for example, by promoting the switch of proinflammatory M1 to regulatory M2 macrophages (8). Consistent with these findings, we found that neutrophil depletion using a Ly6G antibody resulted in an increased and prolonged inflammatory monocyte/macrophage dominated immune response at day 3 after fracture and impaired fracture healing. In contrast, other studies reported improved fracture healing following neutrophil depletion using anti-neutrophil serum or G-CSF-induced neutrophilia (10,11). Mechanistically, neutrophils deposit a fibronectin containing “emergency extracellular matrix” in the early human fracture hematoma (<3 days after fracture), which could serve as a scaffold for mesenchymal stromal cell (MSC) recruitment (12). In this context, neutrophils have also been shown to directly recruit MSCs through the secretion of stromal cell derived factor 1a, thereby orchestrating bone repair (13). The role of neutrophils in bone healing might depend on the severity of trauma, because increased numbers thereof where found in the fracture hematoma following an additional thoracic trauma 24 h after fracture (9,14–18), which is associated with compromised bone healing. On one hand, we found that trauma-induced impaired fracture healing was not affected by neutrophil-depletion, suggesting that neutrophils may become functionally impaired or even “paralyzed” under these conditions. On the other hand, it is well-known that under hyperinflammatory conditions, neutrophils become overactivated, releasing excessive amounts of NETs and inflammatory cytokines (19,20), thereby exacerbating tissue damage and disease progression, for example, in rheumatoid arthritis, diabetes or smoking (21–23). Therefore, hyperactivated neutrophils might also contribute to disruptions in the finely tuned fracture healing process. Indeed, increased neutrophil presence and activity, along with the subsequent release of inflammatory mediator disrupted fracture callus mineralization (24). Therefore, balanced neutrophil activity appears to be crucial for effective fracture healing. However, current knowledge of neutrophil functions and signaling in this process remains preliminary.

Neutrophils are regarded to be a crucial source of interleukin-6 (IL-6) and the soluble IL-6 receptor (sIL-6R) during immune responses (25–29), although they are not the single source of IL-6 as also other immune cells including macrophages, and both B and T cells, secrete IL-6 (30,31). The pleiotropic cytokine IL-6 can exert both pro- and anti-inflammatory effects and plays a critical role in regulating inflammation and bone repair. IL-6 is rapidly released at the fracture site, with peak levels occurring 6 h after fracture (32,33). It is essential for the recruitment and activation of other immune cells that clear debris and release additional signaling molecules (34,35). Furthermore, IL-6 promotes angiogenesis and regulates later stages of fracture healing by influencing chondrocyte and osteoblast differentiation, as well as osteoclastogenesis, thereby controlling bone formation and callus remodeling (34,36,37). Mechanistic studies using global *IL-6* knockout mice have revealed reduced callus mineralization and persistent cartilage during fracture healing (38,39). However, global deletion of *IL-6* causes dysfunctions in the neuronal, hormonal, and blood-forming systems (40–42), which may interfere with bone repair. Furthermore, these studies did not distinguish between the specific functions of the different IL-6 signaling pathways, which may induce diverse effects during bone repair. IL-6 signaling occurs through two separate mechanisms. In IL-6 classic signaling, IL-6 binds to its specific membrane-bound receptor (mIL-6R), which associates with the ubiquitously expressed gp130 protein, forming a signaling complex and inducing intracellular signal transduction (43). Classic signaling is primarily restricted to cells that express the mIL-6R, such as hepatocytes and various immune cells. However, cells that do not express the mIL-6R can still respond to IL-6 through trans-signaling. In this process, IL-6 binds to the soluble IL-6R (sIL-6R), and the resulting complex interacts with gp130 expressed on almost all cells of the body. The sIL-6R is preferentially secreted by innate immune cells, particularly neutrophils, following proteolytic cleavage of the mIL-6R by the zinc metalloprotease a disintegrin and metalloprotease 17 (44). Supporting these findings, our recent data showed that while all analyzed immune cell populations expressed IL-6, neutrophils—second only to B cells—were a major source of locally produced IL-6 and sIL-6R in the early fracture hematoma at 24 h after fracture (29). Additionally, our results revealed that IL-6 classic signaling, but not trans-signaling, is crucial for a balanced immune response by regulating neutrophil infiltration and/or apoptosis in the fracture hematoma. Classic IL-6 signaling also regulates endochondral bone formation and callus remodeling during the later stages of healing (29).

In conclusion, neutrophils, as the first line of cellular defense, play a critical role in initiating essential steps of the bone healing cascade. Furthermore, neutrophils may be a significant source of IL-6 and the sIL-6R locally at the fracture site. Therefore, the aim of this study was to investigate the role of neutrophil-specific IL-6 signaling in fracture healing using mice with a neutrophil-specific deletion of *IL-6* or *IL-6R*.

## MATERIALS AND METHODS

### *In vivo* experimental mouse models

B6.Ly6G(tm2621(Cre-tdTomato)Arte mice (kindly provided by Prof. Matthias Gunzer, University Hospital Essen, Germany) (45), B6.Il6^tm1.1Jho^ mice (kindly provided by Prof. Juan Hidalgo, Universitat Autònoma de Barcelona, Spain) (46), B6.SJL-Il6ra^tm1.1Drew^ mice (JAX stock: #012944, The Jackson Laboratory, Bar Harbor, ME) (48), and C57BL/6J mice (The Jackson Laboratory) were housed in groups of two to five mice under standard rodent conditions with a 12-hour light, 12-hour dark rhythm and water and standard mouse feed (Sniff R/M-H, V1525-300, Ssniff, Soest, Germany) *ad libitum*. B6.Ly6G(tm2621(Cre-tdTomato)Arte mice were crossed with either B6.Il6^tm1.1Jho^ mice to generate a neutrophil-specific deletion of *IL-6* (*IL-6^Ly6GCre^*) or with B6.SJL-Il6ra^tm1.1Drew^ mice to generate a neutrophil-specific deletion of the *IL-6R* (*IL-6R^Ly6GCre^*). Cre negative littermates (*IL-6^fl/fl^* and *IL-6R^fl/fl^*) or C57BL/6J mice served as controls. The mice were on a C57BL/6J background. For genotyping, ear biopsies were used and genomic DNA was extracted using the DirectPCR Lysis Reagent (Viagen Biotech, Los Angeles, CA) including a proteinase K (1:100, Bioline, London, UK) digestion step. PCR was performed as previously described using the following primers (45–47): Catchup_Ctrl1 5′-GAGACTCTGGCTACTCATCC-3′; Catchup_Ctrl2 5′- CCTTCAGCAAGAGCTGGGGAC-3′; Catchup_KI1 5′-ACGTCCAGACACAGCATAGG-3′; Catchup_KI2 5′- GAGGTCCAAGAGACTTTCTGG-3′; Cacthup_KI_WT 5′- GGTTTTATCTGTGCAGCCC-3′; tdTomato_for 5′-GAGTTCATGCGCTTCAAGGT-3′; tdTomato_rev 5′-CTTCAGCTTGGCGGTCTG-3′; IL-6flox_for 5′-CCCACCAAGAACGATAGTC-3′; IL-6flox_rev 5′-GGTATCCTCTGTGAAGTCTC-3′; IL-6Rflox_for 5-′GAAGGAGGAGCCTTGG-3′; IL-6Rflox_rev 5′- AACCATGCCTCTTTGG-3′.

### Study design

First, we investigated whether the neutrophil-specific deletion of *IL-6* or *IL-6R* influences the physiological immune and bone phenotypes of male mice at 12 and 36 weeks of age, establishing a baseline to compare pathologic changes after fracture. Next, we assessed the impact of these deletions on fracture healing in 12-week-old male mice following femur osteotomy. Effects on fracture healing were analyzed 6 h after fracture, when IL-6 levels in the fracture hematoma and systemically peak during the early inflammatory phase, and on day 21, when the fracture is bridged by new woven bone, allowing for quantitative measurement of the fracture healing outcome. Mice were euthanized with an isoflurane overdose and terminal intracardial blood withdraw.

### Flow cytometry

To investigate whether the neutrophil-specific deletion of *IL-6* or *IL-6R* influences innate and adaptive immune cell populations in the bone marrow, spleen, and inguinal lymph nodes, we performed flow cytometry of 12-week-old male, nonfractured mice. Cre negative littermates or wildtype mice served as controls. Inguinal lymph nodes and spleen were explanted and passed through a cell strainer (70 μm). The bone marrow of the femur was obtained by centrifugation (12,300 rpm for 40 s) and resuspended in phosphate-buffered saline. Erythrocyte lysis of spleen and bone marrow samples was performed using lysis buffer of 150 nM NH_4_CL, 1 mM KHCO_3_, and 0.1 mM Na_2_EDTA (all Sigma Aldrich, St. Louis, MO) for 5 min at 37°C. Cells were stained with the following antibodies: F4/80 FITC (1:50, eBioscience, Frankfurt, Germany), Ly6G V450 (1:400, BD Bioscience, Heidelberg, Germany), CD11b Alexa Fluor 700 (1:400), CD3e PE-cyanine7 (1:100), CD4 APC-eFluor 780 (1:200), CD8a APC (1:800), CD19 PE (1:400) (all eBioscience), and IL6R BUV563 (CD126, clone D7715A7, 1:100, BD Bioscience) for 30 min on ice in the dark. For intracellular IL-6 staining, cells were stimulated with 1 μg/mL lipopolysaccharide (LPS, Thermo Fisher, Waltham, MA) over night at 37°C, followed by restimulation with 1 μg/mL LPS and 5 μg/mL Brefeldin A (Biolegend, San Diego, CA) for 4 h at RT. Cell surface markers were stained with the following antibodies: eFluor450-CD11b (1:100, Biolegend) and Spark UV 387-Ly6G (1:100, Biolegend). Afterwards, cells were fixed with the Cyto-Fast Fix/Perm Kit (Biolegend) according to the manucfacturer’s protocol for 20 min at RT. Then, intracellular IL-6 staining was performed with the AF 488 IL-6 antibody (clone MP5-20F3, 1:100, BD Pharmingen, Franklin Lakes, NJ) for 20 min at RT in the dark. Corresponding isotype controls were used. Live-dead discrimination was performed with 7-aminoactinmycin D (1:100) added to the samples prior to the measurement. A BD FACSLyric flow cytometer (BD Bioscience) was used for sample measurement and FlowJo software (10.0.8r1, FlowJo, Ashland, OR) for analysis.

### X-ray imaging

Whole-body x-ray images were obtained from nonfractured mice using a Faxitron device (35 kV, 11 s, Faxitron, Hologic Inc., Marlborough, MA) directly after euthanization.

### Femur osteotomy

To investigate whether the neutrophil-specific deletion of *IL-6* or *IL-6R* affects fracture healing, 12-week-old male mice received an unilateral femur osteotomy, which was created at the diaphysis using a 0.44 mm Gigli-wire saw (RISystem, Davos, Switzerland) and stabilized by a semi-rigid external fixator (RISystem), as described previously (48). Briefly, after muscle separation, an external fixator was attached to the femur with four mini-Schanz screws, followed by osteotomy gap sawing. The muscle and skin were then closed using Vicryl 5-0 (Ethicon, Somerville, NJ) and Resolon 5-0 (Ethicon) sutures. The mice were allowed to move freely in their cages after surgery. Mice were randomly assigned to the different experiments.

### Multiplex cytokine analysis

Cytokine and chemokine concentrations in the blood and the fracture hematoma of all fractured mice were determined 6 h after fracture using a customized mouse Multiplex Cytokine Kit (Thermo Fisher). The fracture hematoma was harvested and lysed in lysis buffer including protease inhibitors as described previously (17). The Pierce BCA Protein Assay Kit (Thermo Fisher) was used to determine the total protein concentrations of the hematoma samples. Serum and fracture hematoma concentrations of the following mediators were analyzed using the Luminex 100 Total System (Bio-Rad Laboratories, Hercules): eotaxin, C-X-C motif chemokine 1 (CXCL1), interferon-γ (IFNγ), IL-1β, IL-10, IL-13, IL-6, leptin, monocyte chemoattractant protein 1 (MCP-1), receptor activator of nuclear factor kappa-B ligand, tumor necrosis factor α (TNFα), and vascular endothelial growth factor α (VEGFα). The IL-6 plasma levels were determined using a mouse IL-6 enzyme-linked immunosorbent assay kit according to the manufacturer’s instructions (R&D systems, Minneapolis, MA). For the hematoma samples, the mediator concentrations were normalized to the determined total protein concentrations.

### Biomechanical testing

To assess the bending stiffness of both intact and fractured femurs, a nondestructive three-point bending test was conducted as previously described (48). For the fractured femurs, the external fixator was carefully removed prior to testing. Both intact and fractured femora were placed in a material testing machine (1454, Zwick, Ulm, Germany), whereby the proximal end of the femur was fixed in the material testing machine using aluminum cylinder and the femur condyle rested unfixed on the distal bending support. An axial load of up to 4 N was applied at the midshaft of the intact femur or the cranio-lateral side of the fracture callus. Load and deflection data were recorded, and bending stiffness was determined from the slope of the load-deflection curve.

### μCT analysis

After biomechanical testing, intact and fractured femurs were fixed in 4% paraformaldehyde for 48 h and scanned using the Skyscan 1172 (Bruker, Kontich, Belgium) μCT system, operating at 50 kV and 200 mA with a voxel resolution of 8 μm. μCT analysis and calibrations were conducted following the American Society for Bone and Mineral Research (ASBMR) guidelines (49) using a CT Analyzer and CT Volume software (both Bruker). Bone mineral density was assessed using two hydroxyapatite phantoms (250 and 750 mgHA/cm^3^) scanned alongside each sample. Thresholds for mineralized tissue were set at 394 mgHA/cm^3^ for trabecular bone and 642 mgHA/cm^3^ for cortical bone (50). The volume of interest (VOI) for trabecular bone in the intact femur was defined as starting 200 μm proximal to the metaphyseal growth plate of the femoral condyle and extending over a total length of 280 μm. For cortical bone, the VOI was positioned at the mid-diaphysis of the femur with a length of 84 μm. In fractured femurs, the VOI encompassed the entire periosteal callus between the two inner pinholes.

### Histomorphometric analysis

Following μCT analysis, intact and fractured femurs were processed for decalcified histology and embedded in paraffin, as previously described (51). Sections (7 μm) of fractured femurs were stained with Safranin-O to determine the relative amounts of bone, cartilage, and fibrous tissue as a percentage of the total callus size. Analysis was performed by light microscopy (Leica DMI6000B) and using Leica LSX software (Leica, Wetzlar, Germany). The region of interest included the entire periosteal callus between the two inner pinholes, encompassing the fracture gap. Osteoblast and osteoclast parameters were assessed in intact and fractured femurs using Toluidine blue- and tartrate resistant acid-phosphatase (TRAP)-stained sections (7-μm paraffin), respectively, following ASBMR guidelines. In intact femurs, osteoblasts and osteoclasts were counted 100 μm proximal to the metaphyseal growth plate in a rectangular area of 650 × 450 μm by light microscopy (Zeiss Axiophot, Carl Zeiss AG, Oberkochen, Germany) and using the Osteomeasure system (v.4.1.0.0, OsteoMetrics, Decatur). In the fracture callus, osteoblasts and osteoclasts were counted in the periostal callus in a rectangular area of 960 × 700 μm as described above. Thereby, osteoblasts were identified in Toluidine blue-stained sections by their attachment to the bone surface and the typical cubic shape. Osteoclasts were identified in TRAP-stained sections by a positive red TRAP staining, more than two nuclei, and direct contact to the bone surface.

### Statistical analysis

The data are presented as bar charts with the mean ± standard deviation and showing all data points. Samples were allocated to the various analyses in a blinded manner. A subset of samples was used for each analysis, as several analyses were mutually exclusive. Data analysis was performed using GraphPad Prism 9 (GraphPad Software, La Jolla). Data were tested for normal distribution with Shapiro-Wilk normality test and subsequently compared using Students *t* test or Kruskal-Wallis test. The level of significance was set at *P* < 0.05. Statistical outliers were identified using the ROUT method (Q = 5%) and subsequently removed from the analysis. The n numbers are indicated in the figure and table legends. The sample size was determined based on the primary outcome parameter, bending stiffness, using a power of 80% and an alpha level of 0.05, as derived from previous studies.

## RESULTS

### Neutrophil-specific deletion of *IL-6* or *IL-6R* did not influence physiologic bone turnover

**Fig. 1:**
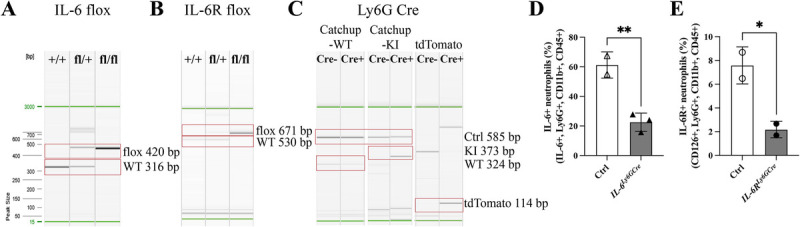
**Mouse genotyping and phenotyping.** PCR-based mouse genotyping showing representative bands identifying (A) IL-6 flox, (B) IL-6R flox, and (C) Ly6G Cre mice. (D) IL-6+ neutrophils (in %) isolated from the bone marrow of control (Ctrl) and *IL-6^Ly6GCre^* mice, and (E) IL-6R+ neutrophils (in %) isolated from the bone marrow of control (Ctrl) and *IL-6R^Ly6GCre^* mice analyzed by flow cytometry. KI, knock-in; PCR, polymerase chain reaction; WT, wildtype. N = 2–3/group, Student’s *t* test, *P < 0.05, **P < 0.01.

We generated mice with a neutrophil-specific deletion of *IL-6* or *IL-6R* by crossing B6.Ly6G(tm2621(Cre-tdTomato)Arte mice with B6.Il6^tm1.1Jho^ or B6.SJL-Il6ra^tm1.1Drew^ mice. The genotypes of the mice were confirmed by PCR (Fig. 1, A–C). Neutrophil-specific deletions were verified at the protein level by flow cytometric analysis, respectively showing fewer IL-6+ and IL-6R+ neutrophils compared to controls (Fig. 1, D and E; Supplemental Fig. 1, http://links.lww.com/SHK/C418; Supplemental Fig. 2, http://links.lww.com/SHK/C418).

**Fig. 2:**
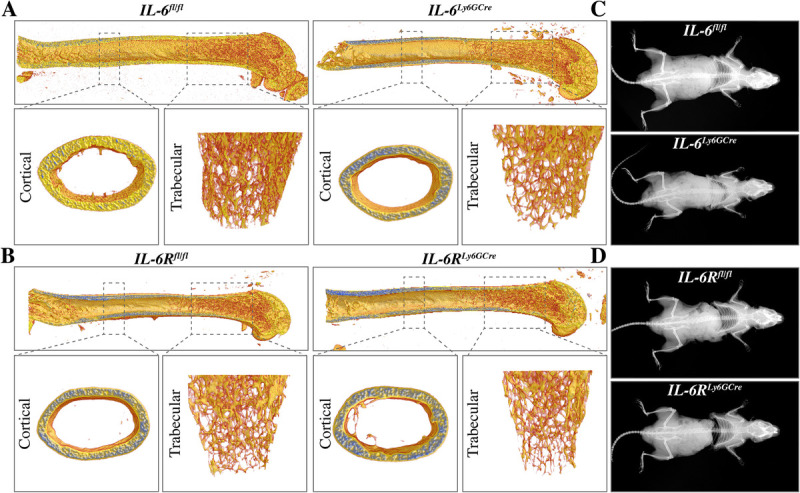
**Physiologic bone phenotype of male mice.** (A and B), Representative μCT reconstructions of the trabecular and cortical bone compartments of the femur of *IL-6^Ly6GCre^* and *IL-6R^Ly6GCre^* mice and their respective littermate controls aged 12 weeks. (C and D), Representative whole-body x-ray images of *IL-6^Ly6GCre^* and *IL-6R^Ly6GCre^* mice and their respective littermate controls aged 36 weeks.

To investigate whether neutrophil-specific deletions of either *IL-6* (*IL-6^Ly6GCre^*) or *IL-6R* (*IL-6R^Ly6GCre^*) affect physiologic bone turnover, the skeleton of male mice aged 12 or 36 weeks was analyzed. In cortical bone of the femora, no differences in bending stiffness, bone mineral density, or cortical thickness were detectable in both *IL-6^Ly6GCre^* and *IL-6R^Ly6GCre^* mice compared to their respective controls (Fig. 2, A and B, Table 1). In trabecular bone, *IL-6^Ly6GCre^* mice displayed a significantly reduced tissue mineral density and trabecular thickness compared to their controls, whereas other parameters did not differ (Table 1). In *IL-6R^Ly6GCre^* mice, no changes in trabecular bone parameters were detected compared to their littermate controls (Table 1). Consistent with these results in trabecular bone, osteoblast and osteoclast parameters did not differ in mice with neutrophil-specific *IL-6* or *IL-6R* deletion compared to their respective controls (Table 1). On the basis of whole-body x-ray images, both male *IL-6^Ly6GCre^* and *IL-6R^Ly6GCre^* mice aged 36 weeks displayed no macroscopic skeletal abnormalities (Fig. 2, C and D). Furthermore, we did not detect differences in the cortical or trabecular bone, and cellular parameters compared to controls (Table 2).

**Table 1:** Bone phenotype of the femur of 12-week-old male mice

Parameters	*IL-6* ***^fl/fl^***	*IL-6* ***^Ly6GCre^***	*IL-6R* ***^fl/fl^***	*IL-6R* ***^Ly6GCre^***
EI (Nmm^2^)	2,489 ± 293	3,033 ± 668	3,350 ± 489	2,847 ± 628
CtTMD (mgHA/cm^3^)	1,244 ± 26	1,177 ± 120	1,145 ± 48	1,131 ± 65
CtTh (mm)	0.094 ± 0.000	0.093 ± 0.001	0.093 ± 0.000	0.093 ± 0.001
TMD (mgHA/cm^3^)	738 ± 27	645 ± 31*	640 ± 15	617 ± 23
BV/TV (%)	11.7 ± 2.8	12.2 ± 4.6	13.0 ± 1.2	11.4 ± 2.2
TbTh (mm)	0.056 ± 0.004	0.046 ± 0.004*	0.047 ± 0.001	0.044 ± 0.003
TbN (1/mm)	2.09 ± 0.49	2.62 ± 0.81	2.74 ± 0.20	2.60 ± 0.59
TbSp (mm)	0.200 ± 0.020	0.164 ± 0.042	0.181 ± 0.005	0.158 ± 0.040
NOb/BPm (1/mm)	12.6 ± 5.2	13.9 ± 3.2	12.7 ± 3.4	12.6 ± 3.3
ObS/BS (%)	8.4 ± 1.9	8.8 ± 2.2	7.5 ± 1.7	8.4 ± 2.4
NOc/BPm (1/mm)	2.88 ± 1.93	4.89 ± 1.31	5.39 ± 1.36	5.19 ± 1.72
OcS/BS (%)	5.05 ± 2.73	7.74 ± 1.94	9.14 ± 1.80	7.74 ± 2.17

*P < 0.01 compared to *IL-6^fl/fl^* or *IL-6R^fl/fl^*, respectively. Student’s *t* test, n = 3–7/group.

BV/TV, bone volume per tissue volume; CtTh, cortical thickness; CtTMD, cortical tissue mineral density; EI, bending stiffness; NOb/BPm, number of osteoblasts per bone perimeter; NOc/BPm, number of osteoclasts per bone perimeter; ObS/BS, osteoblast surface per bone surface; OcS/BS, osteoclast surface per bone surface; TbN, trabecular number; TbSp, trabecular separation; TbTh, trabecular thickness; TMD, tissue mineral density.

**Table 2:** Bone phenotype of the femur of 36-week-old male mice

Parameters	*IL-6* ***^fl/fl^***	*IL-6* ***^Ly6GCre^***	*IL-6R* ***^fl/fl^***	*IL-6R* ***^Ly6GCre^***
EI (Nmm^2^)	3,633 ± 646	3,211 ± 519	3,801 ± 606	4,130 ± 521
CtTMD (mgHA/cm^3^)	1,371 ± 29	1,343 ± 60	1,348 ± 72	1,222 ± 21
CtTh (mm)	0.094 ± 0.000	0.093 ± 0.000	0.093 ± 0.000	0.093 ± 0.000
TMD (mgHA/cm^3^)	734 ± 35	734 ± 33	752 ± 33	689 ± 45
BV/TV (%)	8.5 ± 1.62	9.3 ± 1.8	11.6 ± 2.2	11.3 ± 5.0
TbTh (mm)	0.061 ± 0.004	0.057 ± 0.003	0.057 ± 0.004	0.054 ± 0.007
TbN (1/mm)	1.41 ± 0.32	1.61 ± 0.25	2.03 ± 0.39	2.08 ± 0.72
TbSp (mm)	0.230 ± 0.029	0.213 ± 0.015	0.209 ± 0.007	0.205 ± 0.018
NOb/BPm (1/mm)	7.7 ± 3.7	5.8 ± 2.1	7.1 ± 4.2	5.8 ± 2.0
ObS/BS (%)	7.0 ± 3.1	6.1 ± 1.5	5.8 ± 2.4	5.6 ± 1.7
NOc/BPm (1/mm)	0.90 ± 0.84	1.69 ± 1.31	2.28 ± 1.02	1.63 ± 1.13
OcS/BS (%)	1.69 ± 1.23	3.51 ± 2.91	4.94 ± 2.69	3.26 ± 1.97

N = 5–7/group.

BV/TV, bone volume per tissue volume; CtTh, cortical thickness; CtTMD, cortical tissue mineral density; EI, bending stiffness; NOb/BPm, number of osteoblasts per bone perimeter; NOc/BPm, number of osteoclasts per bone perimeter; ObS/BS, osteoblast surface per bone surface; OcS/BS, osteoclast surface per bone surface; TbN, trabecular number; TbSp, trabecular separation; TbTh, trabecular thickness; TMD, tissue mineral density.

In conclusion, the neutrophil-specific deletion of *IL-6* did not influence the physiologic bone phenotype of male mice, except for minor changes observed in tissue mineral density and trabecular thickness at the age of 12 weeks. Additionally, *IL-6R* deletion in neutrophils did not affect the physiologic bone phenotype of male mice at either age. These results suggest that neither IL-6 nor IL-6R signaling in neutrophils plays an essential role in physiologic bone turnover.

### Neutrophil-specific deletion of *IL-6* did not influence physiologic immune phenotype

**Fig. 3:**
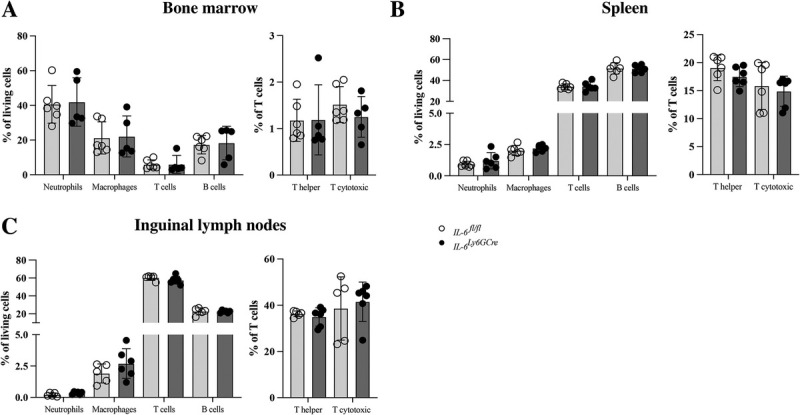
**Immune cell populations of 12-week-old *IL-6^fl/fl^* and *IL-6^Ly6GCre^* male mice.** Percentages of neutrophils, macrophages, T cells, B cells (all % of living cells), T helper cells, and T cytotoxic cells (% of T cells) in the (A) bone marrow, (B) spleen, and (C) inguinal lymph nodes. n = 5–6/group.

Because neutrophils cross-talk with other immune cells, we investigated whether the specific deletion of *IL-6* in neutrophils affected the different immune cell populations in the bone marrow, spleen, and inguinal lymph nodes of nonfractured mice (Supplemental Fig. 3, http://links.lww.com/SHK/C418). *IL-6^Ly6GCre^* mice displayed no differences in the percentages of macrophages, neutrophils, T cells, or B cells in the investigated organs compared to the littermate controls (Fig. 3, A–C). Furthermore, the percentages of T helper and T cytotoxic cells did not differ compared to controls (Fig. 3, A–C). These results indicated that IL-6 secreted from neutrophils does not influence other immune cell populations under physiologic conditions.

### Neutrophil-specific deletion of *IL6R* significantly affected physiologic immune phenotype

**Fig. 4:**
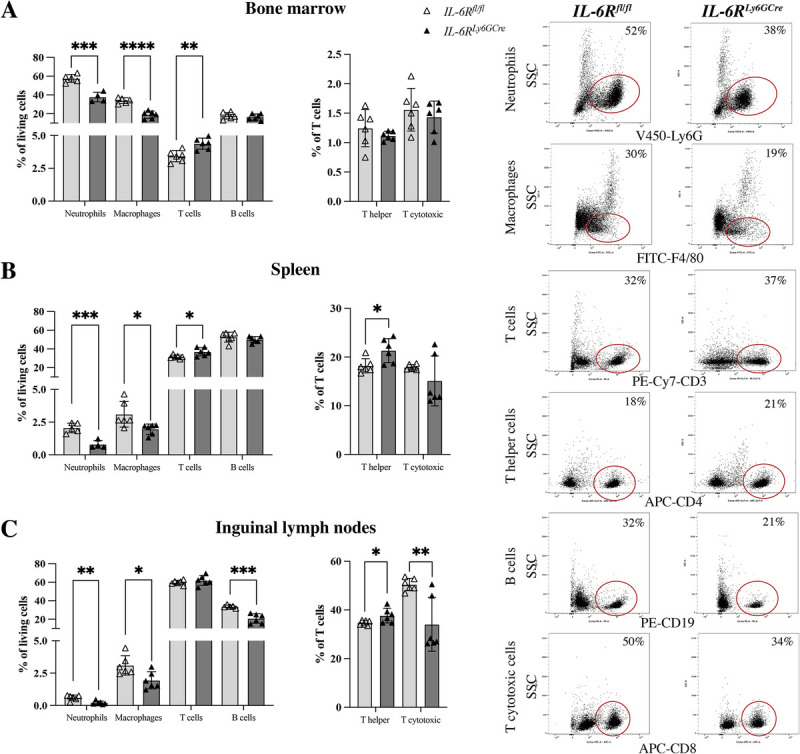
**Immune cell populations of 12-week-old *IL-6R^fl/fl^* and *IL-6R^Ly6GCre^* male mice.** Percentages of neutrophils, macrophages, T cells, B cells (all % of living cells), T helper cells, and T cytotoxic cells (% of T cells) in the (A) bone marrow, (B) spleen, and (C) inguinal lymph nodes. Representative dot plots of CD11b+/Ly6G+ neutrophils and CD11b+/F4/80+ macrophages in the bone marrow; CD3+ T cells and CD3+/CD4+ T helper cells in the spleen; and CD19+ B cells and CD3+/CD8+ T cytotoxic cells in the inguinal lymph nodes. n = 4–6/group, Students *t*-test, *P < 0.05, **P < 0.01, ***P < 0.001, *P* < 0.0001.

Subsequently, we investigated whether the neutrophil-specific deletion of *IL-6R* affects immune cell populations in nonfractured mice (Supplemental Fig. 3, http://links.lww.com/SHK/C418). Indeed, in the bone marrow, spleen, and inguinal lymph nodes, the percentages of neutrophils and macrophages were significantly reduced in *IL-6R^Ly6GCre^* compared to *IL6-R^fl/fl^* mice (Fig. 4, A–C). Furthermore, the percentage of T cells was significantly increased in the bone marrow and spleen of *IL-6R^Ly6GCre^* mice, with no changes detectable in inguinal lymph nodes (Fig. 4, A, B). The percentage of B cells was significantly reduced in inguinal lymph nodes of *IL-6R^Ly6GCre^* mice compared to littermate controls (Fig. 4, C), but not in the bone marrow or spleen. Moreover, we detected significant changes in the percentages of T helper and T cytotoxic cells in *IL-6R^Ly6GCre^* mice. In the spleen, more T helper cells were found in comparison to littermate controls (Fig. 4). In inguinal lymph nodes, *IL-6R^Ly6GCre^* mice displayed an increased percentage of T helper cells, whereas the percentage of T cytotoxic cells was significantly reduced compared to littermate controls (Fig. 4). In conclusion, neutrophil-specific deletion of *IL-6R* significantly affected immune cell populations under physiologic conditions.

### Neutrophil-specific deletion of *IL-6* or *IL-6R* did not influence the immune response after fracture

To investigate whether IL-6 signaling in neutrophils influences the early immune response upon fracture, male 12-week-old *IL-6^Ly6GCre^* and *IL-6R^Ly6GCre^* mice, together with their respective littermate controls, underwent a standardized femur osteotomy stabilized by an external fixator. Pro-inflammatory cytokine and chemokine concentrations were determined both systemically in the blood and locally in the fracture hematoma after 6 h. Surprisingly, we did not detect differences in the systemic levels of eotaxin, CXCL-1, IL-1β, IL-6, or TNFα in either of the mice compared to their controls (Table 3). Furthermore, the local concentrations of eotaxin, CXCL-1, IFNγ, IL-1β, IL-10, IL-6, MCP1, TNFα, and VEGFα in the fracture hematoma did not differ between the mice with neutrophil-specific deletions of *IL-6* or *IL-6R* and their respective controls (Table 3). In conclusion, IL-6 signaling in neutrophils does not appear to regulate the immune response following fracture, suggesting that neutrophils are not a critical source of IL-6 during fracture healing.

**Table 3:** Inflammatory mediator concentrations in the blood and fracture hematoma of mice 6 h after fracture

Cytokine	*IL-6* ***^fl/fl^***	*IL-6* ***^Ly6GCre^***	*IL-6R* ***^fl/fl^***	*IL-6R* ***^Ly6GCre^***
**Blood (pg/mL)**				
Eotaxin	188 ± 67	134 ± 79	215 ± 96	151 ± 80
CXCL-1	782 ± 748	1,123 ± 1,067	1791 ± 679	1,149 ± 431
IL-1β	4.10 ± 3.94	1.29 ± 1.86	0.5 ± 1.2	2.4 ± 5.3
IL-6	3.43 ± 2.25	2.94 ± 1.00	190 ± 192	50 ± 36
TNFα	2.54 ± 5.69	2.23 ± 4.98	13.5 ± 10.8	3.7 ± 8.4
**Fracture hematoma (pg/mg total protein)**				
Eotaxin	10.4 ± 5.7	8.6 ± 5.7	30.9 ± 38.7	18.9 ± 20.6
CXCL-1	369 ± 221	343 ± 260	366 ± 285	444 ± 233
IFNγ	1.12 ± 0.42	0.81 ± 0.33	1.22 ± 0.82	1.44 ± 0.22
IL-1β	13.7 ± 6.1	27.3 ± 38.7	15.6 ± 15.0	16.0 ± 13.8
IL-10	9.60 ± 4.72	7.51 ± 5.39	20.6 ± 13.5	20.1 ± 2.9
IL-6	664 ± 373	482 ± 286	2,891 ± 2,166	2,276 ± 1,029
MCP1	342 ± 202	394 ± 278	348 ± 288	355 ± 158
TNFα	11.5 ± 6.3	11.5 ± 7.8	35.1 ± 21.9	34.4 ± 12.1
VEGFα	40.5 ± 13.2	41.0 ± 18.6	30.5 ± 28.2	32.6 ± 32.6

N = 5–6/group.

CXCL1, C-X-C motif chemokine ligand 1; IFNγ, interferon γ; IL, interleukin; TNFα, tumor necrosis factor α; MCP1, monocyte chemoattractant protein 1; VEGFα, vascular endothelial growth factor α.

### Neutrophil-specific deletion of *IL-6* or *IL-6R* did not influence bone formation after fracture

**Fig. 5:**
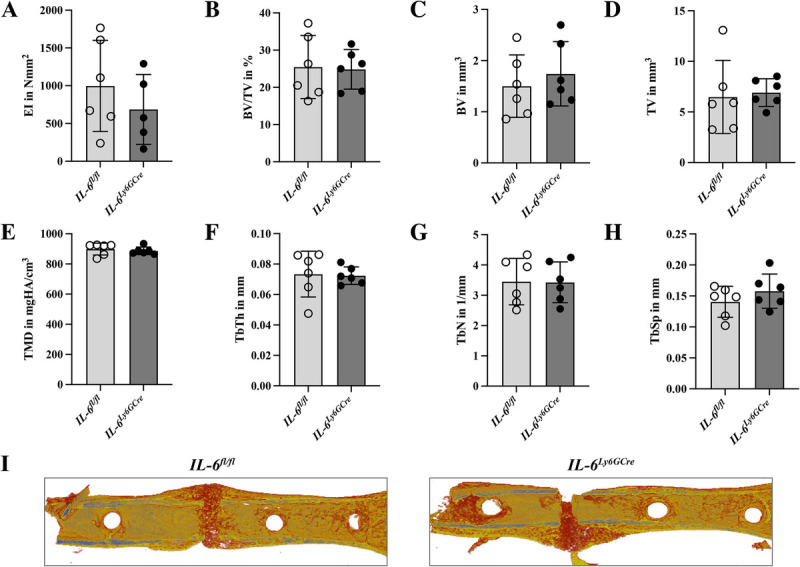
**Bone formation at day 21 after fracture in the callus of *IL-6^fl/fl^* and *IL-6^Ly6GCre^* mice.** (A) Bending stiffness (EI), (B) BV/TV, (C) BV, (D) TV, (E) TMD, (F) TbTh, (G) TbN, (H) TbSp, and (I) representative 3D-reconstructions of the fracture callus. n = 6/group. EI, bending stiffnes; BV, bone volume; BV/TV, bone volume per tissue volume; TbN, trabecular number; TbSp, trabecular separation; TbTh; trabecular thickness; TMD, tissue mineral density; TV, tissue volume.

At day 21 after fracture, we investigated whether neutrophil-specific deletion of *IL-6* or *IL-6R* affect bone formation and healing outcomes. In *IL-6^Ly6GCre^* mice, the bending stiffness of the fractured femurs (Fig. 5A), as well as the structural parameters of the fracture callus—including tissue mineral density, bone volume fraction, bone volume, tissue volume, and trabecular parameters such as thickness, number, and separation (Fig. 5, B–I)—did not differ compared to littermate controls.

**Fig. 6:**
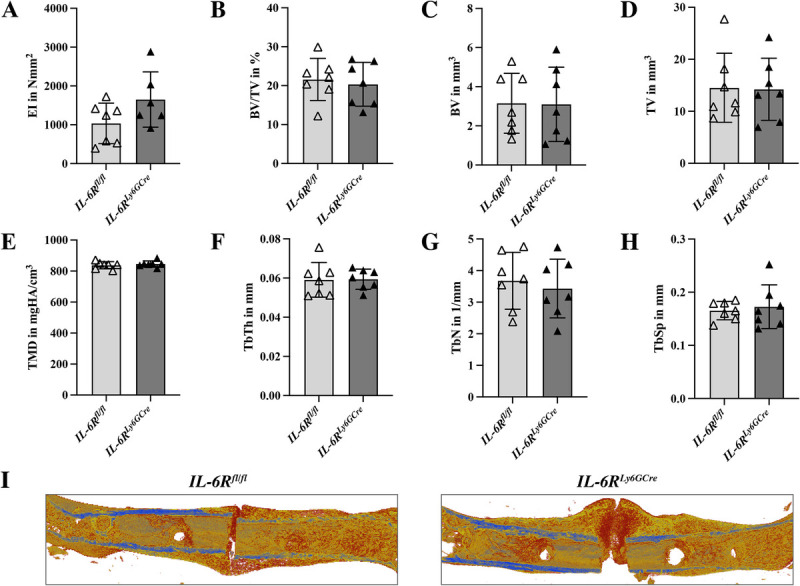
**Bone formation at day 21 post-fracture in the callus of *IL-6R^fl/fl^* and *IL-6R^Ly6GCre^* mice.** (A) Bending stiffness (EI), (B) BV/TV, (C) BV, (D) TV, (E) TMD, (F) TbTh, (G) TbN, (H) TbSp, and (I) representative 3D-reconstructions of the fracture callus. n = 6–7/group. EI, bending stiffness; BV, bone volume; BV/TV, bone volume per tissue volume; TbN, trabecular number; TbSp, trabecular separation; TbTh; trabecular thickness; TMD, tissue mineral density; TV, tissue volume.

Additionally, in *IL-6R^Ly6GCre^* mice, we did not detect any differences in the bending stiffness (Fig. 6A), mineralization, or structural composition of the fracture callus compared to their littermate controls (Fig. 6, B–I).

**Fig. 7:**
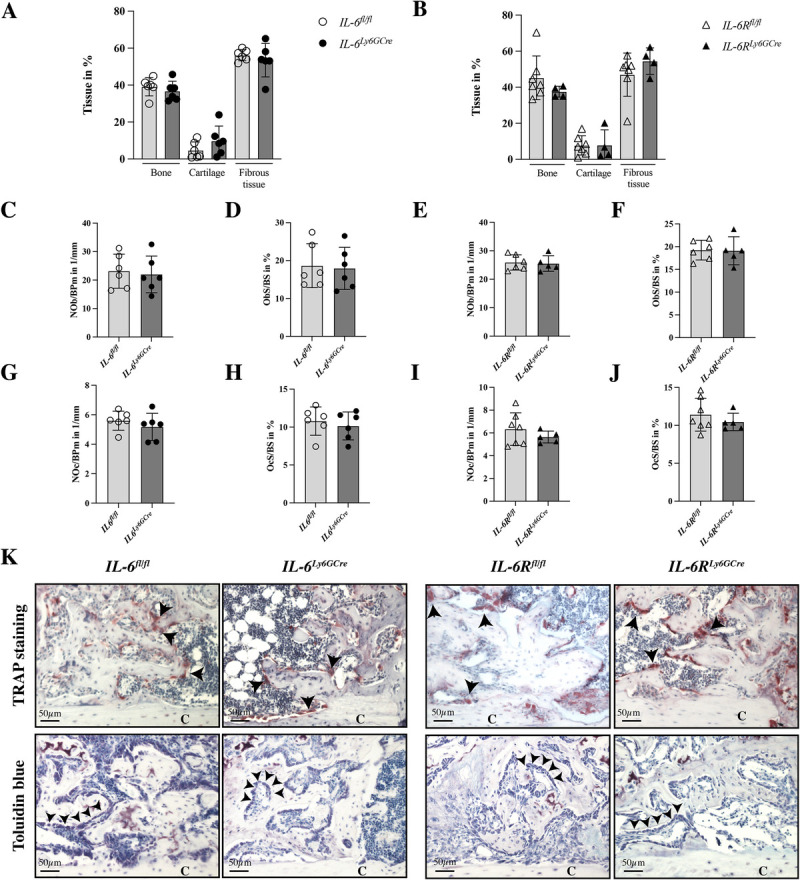
**Callus composition at day 21 post-fracture of *IL-6^Ly6GCre^* and *IL-6R^Ly6GCre^* mice.** (A and B), Fracture callus tissue composition (%). (C), NOb/BPm and (D) ObS/BS of *IL-6^Ly6GCre^* mice. (E), NOb/BPm and (F) ObS/BS of *IL-6R^Ly6GCre^* mice. (G), NOc/BPm, and (H) OcS/BS of *IL-6^Ly6GCre^.* (I), NOc/BPm and (J) OcS/BS of *IL-6R^Ly6GCre^* mice. (K), Representative images of TRAP- (osteoclasts indicated by arrowheads), and Toluidine blue- (osteoblasts indicated by arrowheads) stained fracture calli at day 21. n = 4–7/group. NOb/BPm, number of osteoblasts per bone perimeter; NOc/BPm, number of osteoclasts per bone perimeter; ObS/BS, osteoblast surface per bone surface; TRAP, tartrate-resistant acid phosphatase.

Consistent with these findings, histomorphometric analysis revealed no significant differences in the relative amounts of bone, cartilage, or fibrous tissue in both, *IL-6^Ly6GCre^* and *IL-6R^Ly6GCre^* mice compared to their littermate controls (Fig. 7, A and B). Similarly, in the fracture callus, no significant changes were observed in the number of osteoblasts per bone perimeter or in the osteoblast surface per bone surface (Fig. 7, C–F and K) in both mice. In addition, osteoclast parameters did not differ between the groups (Fig. 7, G–J and K). In conclusion, IL-6 signaling in neutrophils does not appear to regulate bone formation during fracture healing.

## DISCUSSION

The present study elucidated the role of neutrophil-derived IL-6 signaling during fracture healing using neutrophil-specific *IL-6* or *IL-6R* knockout mice. First, we examined the bone phenotype of these mice under normal (physiologic) conditions. While physiologic bone turnover was unaffected by either deletion, *IL-6R* deletion in neutrophils significantly altered immune cell populations in several immune-related organs. Despite this, neither the immune response to fracture, including IL-6 levels, nor bone repair were significantly impacted by the specific deletion of *IL-6* or *IL-6R* in neutrophils, suggesting that neutrophil-generated IL-6 signaling plays a subordinate role in regular fracture healing and that neutrophils are not the major source for IL-6 under these conditions.

Fracture healing starts with an inflammatory phase, initially dominated by neutrophils and elevated cytokine levels, including IL-6, which both play important roles in orchestrating the early and late repair processes (1,36,52). Neutrophils clear cell and tissue debris, release NETs and ROS, and secrete a range of proteases and proinflammatory mediators. In addition, neutrophil-derived growth factors and cytokines play key roles in the polarization of macrophages to the M1 or M2 phenotype (8,53,54), promote angiogenesis, and facilitate the recruitment of MSC (55). These progenitor cells become embedded within the fibrin-rich extracellular matrix released by neutrophils, thereby promoting bone repair (12). On the other hand, increased neutrophils numbers, along with prolonged and excessive activation, have been associated with impaired fracture healing (9,24). However, the underlying cellular and molecular mechanisms remain incompletely understood. As in 5%–10% of patients, healing is delayed or fails altogether, there is an urgent need for new therapeutic options to support these patients (56,57). Identification of the molecular and cellular key mechanisms involved is crucial for developing targeted therapeutic strategies. Our recent data indicated that neutrophils are a source of IL-6 and the sIL-6R after fracture, and that IL-6 classic signaling regulates the fracture healing process (29). Here, we aimed to investigate whether IL-6 secreted from neutrophils is directly responsible for these proregenerative effects. To address this, we generated mice with a neutrophil-specific deletion of *IL-6* or *IL-6R*.

We first examined whether this knockout impacted the physiologic bone phenotype, which is crucial for establishing a baseline to compare pathologic changes after fracture. Both deletions did not alter physiological bone turnover, indicating that neutrophil-induced IL-6 signaling is unimportant for the regulation of bone homeostasis. By contrast, mice depleted of neutrophils exhibited a significantly reduced bone mass by directly inhibiting osteoblastogenesis (58). Supporting our findings of unchanged baseline bone parameters, mice without tissue trauma or an inflammatory stimulus do not express significant amounts of IL-6 in their circulation (48). Furthermore, deletion of *IL-6* from neutrophils did not alter immune cell populations in several immune-related organs. By contrast, *IL-6R* deletion from neutrophils significantly reduced neutrophil and macrophage populations while increasing T lymphocyte levels in the bone marrow and spleen under physiologic conditions. These results may have several implications: A) It is possible that classical IL-6 signaling in neutrophils, which requires the mIL-6R, is essential for neutrophil survival. Indeed, previous studies have shown that blocking classical IL-6 signaling reduces neutrophil survival and migration during acute liver injury (59), highlighting the importance of this pathway in these cells. B) It is also reasonable to assume that the sIL-6R, which is shed from the neutrophil surface, might play a critical role in the survival, activation, and/or polarization of other immune cell types, such as macrophages and T lymphocytes. Previous studies have demonstrated that blocking IL-6 trans-signaling inhibits T lymphocyte polarization into the gamma-delta (γδ) T cell during airway inflammation, which could explain the increased number of unpolarized T cells observed in immune organs in our study (60). Furthermore, studies showed that blocking IL-6 trans-signaling reduces macrophage numbers in a mouse model of sepsis (61) and that IL-6 trans-signaling plays a crucial role in MCP-1 upregulation in immune-mediated myopathy (62). IL-6 trans-signaling plays a significant yet also controversial role, depending on the tissue and model used, in various murine tissue trauma models (61,63–66). C) Another possibility is that neutrophils lacking *IL-6R* expression have an altered secretome, which may indirectly influence other immune cell types within their niches in the bone marrow or spleen. However, this needs to be investigated in future studies. To limit our study, we did not further characterize neutrophils using CD45-based purification followed by flow cytometry or additional markers. Additionally, we did not assess whether IL-6 or IL-6R knockout might have distinct effects on neutrophil subtypes, including N1 or N2 subsets. Moreover, assessing whether the deletions affect neutrophil functions, such as NET formation, phagocytosis, and apoptosis, would be important but was beyond the scope of our study. These aspects should be explored in future research. Concluding our results, the IL-6R on neutrophils appears to play a crucial role in maintaining immune homeostasis under physiologic conditions.

However, when investigating fracture healing, which involves activation of both neutrophils and IL-6 signaling due to tissue trauma, we did not detect any differences between our knockout and control mice. This suggests: A) Neutrophils are not the primary cellular source of IL-6 during fracture healing. B) Neither the classical IL-6 signaling pathway in neutrophils nor the sIL-6R shed by neutrophils are critically involved in the fracture healing process (17,29). Regarding A), we hypothesize that mast cells, rather than neutrophils, may be one of the main cellular sources of IL-6 during fracture healing. This is based on our previous findings that mast cell-deficient mice have reduced IL-6 levels in both, the circulation and the hematoma after fracture (32). Additionally, mast cells have been demonstrated to store large amounts of IL-6 in their granules and can rapidly release this cytokine upon activation by inflammatory stimuli (67). However, this hypothesis requires further investigation, such as by generating mast cell-specific *IL-6* knockout mice. Additionally, several other cell types, including macrophages, B and T cells, and osteoblasts also secrete IL-6 (30,31). It is possible that IL-6 from these cell types is more crucial for fracture healing, or that IL-6 released from these cells compensated for the lack of IL-6 in neutrophils. Furthermore, IL-6 deletion in neutrophils was nor complete, as 20% of neutrophils still expressed IL-6, indicating that the recombination did not occur in all cells, which could be attributed to the used Cre model. The residual IL-6 may have been sufficient to sustain necessary biological responses during fracture healing. Regarding B), the absence of an effect on fracture healing in *IL-6R* knockout mice was surprising, because we had already observed immune cell changes under baseline conditions in these mice. One possible explanation is that the role of the IL-6R on neutrophils may depend on trauma severity. Previous studies have shown that blocking classical IL-6 signaling negatively affects fracture healing in a single-injury model, whereas blocking IL-6 trans-signaling prevents delayed fracture healing in a double-injury model with hyperinflammation induced by fracture and thoracic trauma (17,29). Although neutrophils themselves may be paralyzed in the combined trauma model, as shown by our previous study (9), their increased presence could lead to elevated IL-6R expression and shedding of the soluble IL-6R, potentially influencing fracture healing. Further investigating the healing response of neutrophil-specific *IL-6R* knockout mice in a double-injury scenario would, therefore, be of interest, but was beyond the scope of our study. Furthermore, different neutrophil subpopulations are known to exist during fracture healing, with N1 and N2 neutrophils potentially having opposing roles in inflammatory responses (68). Consequently, deleting the IL-6R across all neutrophils might result in compensatory effects, where negative impacts are balanced by more positive outcomes. Unfortunately, no Cre models are available to enable gene deletion specifically in neutrophil subpopulations, thereby preventing direct testing of this hypothesis *in vivo* at present. Distinguishing between N1 and N2 neutrophil subsets in mice remains an evolving area, particularly in the context of fracture healing, as universally accepted markers for these subsets are still lacking. Nevertheless, exploring the complex interplay between neutrophils subsets warrants further future investigation, given their diverse roles during inflammation. This can be achieved through flow cytometry combining surface markers with intracellular stainings or by evaluating transcription factors and cytokine gene expression by qPCR or RNA-Sequencing. In addition, it would be interesting to investigate whether neutrophil-specific IL-6 signaling plays a role in aged mice, as aging is associated with increased inflammation, cellular senescence, mitochondrial dysfunction, and immune system dysregulation (69). Previous studies have shown that aging impairs fracture healing, with notable dysregulations in macrophage subtypes and impaired vascularization and angiogenesis (70–72). Furthermore, neutrophils in aged mice have been shown to be functionally compromised (73). Therefore, the effects on fracture healing might be more pronounced in aged mice, which could be an important investigation for future research.

Nevertheless, our model has also limitations. We used an external fixator for fracture stabilization, although it is less commonly applied in clinical practice. Nonetheless, is a well-established and widely used method in murine fracture models, allowing standardized stable fracture fixation, minimal soft tissue disruption, and the ability to study bone healing under controlled mechanical conditions (74). While there are limitations in directly translating findings from murine models to humans (75), animal studies remain essential for understanding fundamental mechanisms of bone healing and for preclinical testing of therapeutic strategies. Using a well-established murine fracture healing model, our study contributes to a deeper understanding of the mechanisms regulating this complex process. Murine models, in particular, provide valuable insights, due to the high degree of similarity in bone cell biology between mice and humans, as well as well characterized skeletal phenotypes. Furthermore, the ability to manipulate the genome of mice allows the investigation of specific gene functions (76).

In conclusion, we demonstrated that neutrophil-specific *IL-6* or *IL-6R* deletion does not affect the physiologic bone phenotype or fracture healing, although *IL-6R* deletion led to alterations in immune cell homeostasis. Given our hypothesis that neutrophil-derived IL-6 signaling may depend on trauma severity, it would be valuable to repeat these experiments under more challenging conditions, such as in a double-injury or polytrauma model characterized by hyperinflammation. This warrants further investigation.
